# Preventive Dentistry—Prophylaxis

**Published:** 1910-10-15

**Authors:** Alice G. Harvie Duden

**Affiliations:** Indianapolis, Indiana


### PREVENTIVE DENTISTRY—PROPHYLAXIS.

BY ALICE G. HARVIE DUDEN, D.D.S., INDIANAPOLIS, INDIANA.

A dentist confided to me the other day the fact that prophylaxis was rather getting on his nerves. He said he felt he could hardly get away from it, turn where he would, it almost pursued him, scarce could he find a dental journal these days but what had one or several articles devoted to it. Several colleges that he knew of, had devoted each a chair to that branch (why hadn't they thought of it when he was at dental college?) and two specialists were devoting most of their time to it in Indianapolis and seemed overwhelmed with work; they never seemed to have a quiet day, such as he himself had once in awhile—and only this last week two of his very best patients (he called them his STAR patients), had been in to ask if he couldn't give them some prophylaxis. They said they had some friends who were having prophylaxis treatment regularly, and as a result expected to have little or no more decay, and their mouths were beginning to look perfectly beautiful—that they themselves would like to have some prophylaxis, but didn't want to leave him if he could give them the treatment, as they had been his patients for so many years. He was very frank with me and said had it been some others of his patients he might have bluffed them a little—cleaned up their teeth and told them they needn't worry, their mouths were all right—but he said these were two very intelligent people —they had gotten hold of some literature on the subject and were pretty well informed. He had to tell them he had not been doing it—and then they rather wondered why. He had no good reason to give, and told them frankly that if they were bent on having preventive dentistry (prophylaxis), they had better go to Dr. Blank, who was giving it, that inasmuch as he had not taken it up he wasn't going to experiment on them.

He confided to me that they were people whom he respected highly, and he knew they would still think a whole lot more of him even if he confessed to not practicing it rather than if he tried to mislead them. They thanked him, expressed appreciation of his most honest advice, but, to his mortification he saw those two patients (they were together) marching their way to Dr. Blank's office. He said he had a fit of what is commonly called blue-funk. He knew in his heart that his patients had left his office through no one's fault but his own. He had at least been honest, his conscience was clear—but as for those two patients, if they were going for a course of preventive dentistry, well, the future for him, so far as they were concerned, didn't look very bright—all he had to offer them was restoration, nothing else—it was all he had been taught.

I comforted him as best I could, telling him that his outlook was most hopeful—inasmuch as he had reached that stage where he was keenly alive to the demands of prophylaxis—and that the insistence with which it demanded recognition was marvelous.

This particular man whom I have referred to represents but one of many in his class. There is a desire to give prophylaxis treatment because it is up-to-date, representing as it does the most advanced ideas in dentistry; and the best of the thinking people of the community are calling for it. There is, however, a better reason than that of simply being up-to-date, which requires prophylaxis from us. When the magnitude of the noble work has been grasped by one who will give a little time and thought to the matter it requires the stifling of a very painful conscience not to offer it to one's patients. The matter is a grave, a serious one,—let us look it squarely in the face. Have we the right to intimate to patients that they shall have no other service than that of restoration? I ask by what right shall we deny our patients *the best*, which is that of prevention of caries and all other dental troubles? Is it not, upon reflection, a cause for keen regret when we acknowledge to ourselves, our patients and to the world at large, the fact that we have little but mechanics to offer; that *patching* (whether it be filling, a crown, a bridge, or a plate), is the aim and end of our efforts; that a patient presenting a cavity, a loss of teeth, or an edentulous jaw—brings joy to us, for we'll make a nice crutch for him to get along with for a while—we will make a piece of something to imitate—a next best, why shall we not offer to him instead, *the best* —namely the conserving and preserving of his teeth to him forever; splendidly healthy gums; sanitary mouths. Why shall we not, when it is so comparatively easy? Give me one good reason and I will listen to you—it's beyond my comprehension, the unwillingness of any dentist to have the earnest desire in his heart to at least *offer* such conditions to his patients.

Do I hear someone say, "well what are we going to do for a living if we quit patching? Why! Give prevention, to be sure—and in maintaining and giving prevention to all of your patients you'll be kept so busy you'll be overwhelmed; you'll be kept much busier than you are now, if that be possible. I now some of you are awfully busy men; what I mean is, there will be even greater demands upon your time than you have at present, should you, for instance, undertake to prevent decay and conserve the teeth of all of your present patients; for please bear in mind that prophylaxis does not mean "*one*," "*two*" or "*three*" treatments; it means at least ten treatments a year, once a month for at least ten months of the year. Will patients come? Oh, yes, of course they'll come—try them with a few treatments and then see if they are willing to go back to old methods. I know from a good deal of experience that they will not. You don't ask your patients to come with the fixed idea that they are not going to respond—instead you sort of take it for granted when you tell them what you want them to enjoy—and they'll come; you all, *everyone* of you, have some patients in your practice who would bless you for this privilege—mothers who will make all sorts of sacrifices in order that their children may be spared what they themselves have gone through; those of us who have been doing this work know whereof we speak.

Let me tell you of what a joyful practice I have. I am busy all day long with such a happy set of people—can you imagine a large practice composed principally of patients who have absolutely nothing the matter with their mouths—of little children being glad to come, of a dental chair having no terror for old or young—a short, pleasant sitting, lasting anywhere from thirty to sixty minutes, and the patient carrying away an appointment card bearing the time set for next month's treatment? Of course, in addition to this there are the pyorrhea cases of which I have many—but it's only a short time ere they are made well and eventually are placed on the prophylaxis list. And speaking of pyorrhea, how easy it is to cure and how much easier it is to prevent. How do we prevent it? Why, simply by prophylaxis. How do we cure it? By a simple extension of prophylaxis methods—for pyorrhea disappears before prophylaxis as the dew before the sun. Let us take a closer view of just what oral prophylaxis is, and how prevention in its fullest sense is brought about. You have of course, made observation of the fact that self-cleansing surfaces of tooth structure never decay, i. e., barring natural defects in the enamel, pits, and so forth; in observing this fact you have the keynote to the solution, for prophylaxis aims to keep those parts of the tooth which are involved in the interproximal spaces (the un-get-at-able parts) just as highly polished, just as free from accumulations, secretions, deposits, etc., as the flat or self-cleaning surfaces. If we allow a patient to come less frequently than the regular monthly visit it is more than probable that decalcification has begun at some point due to lactic acid fermentation brought about by the non-removal of carbohydrates, i. e., particles of sugar and starches. No patient can thoroughly remove these deposits and accumulations himself—we must have co-operation—for without it, we will avail little.

I would here remind you that the average mouth possesses anywhere from twenty to thirty square inches of tooth surface; the patient can only successfully care for about two-thirds of this area—it is the other third, the un-get-at-able third that we work especially for—which we render highly lustrous and polished—so that no accumulations can find a foothold. This is the secret.

Could you see magnified the enamel of an average tooth, which has received *no prophylaxis treatment*, you would observe that it is coarse grained—a sort of sandpaper surface, which would offer an ideal foothold for all sorts of deleterious matter, including lactic acid which is the actiological factor in caries. Then if you view in the same manner the enamel of a tooth which has received a course of prophylaxis treatment, you would find it close grained, fine, dense, resistant to acid fluids and very brilliant, which polished surface offers no lodging place to the foes of the denture.

Have I made my point clear, I wonder? This leads me to a phase I must discuss with you. I wish I might pass it over, but my conscience won't allow me to, and that is what is commonly called *cleaning teeth.* The average operation of this kind consists of fifteen or twenty minutes work; a small brush or rubber cup loaded with pumice revolving on the engine, and then the patient is handed a mirror and told to look at the shining appearance of his front teeth. Poor patient, he does not know that the inbetween places, those vulnerable parts, where decay inevitably begins, have not been polished, that they are still loaded with decomposing food, collections and bacteria; he doesn't know that the distal surface of that third molar which may or may not have a fine piece of bridge work anchored to it, is in danger of a pyorrhea pocket or caries, and that his breath is still heavy due to the infectious matter not having been removed. Such a cleaning is, to use harsh words and call a spade a spade, a delusion, a deception, and it reminds me of nothing so forcibly as the dirty school boy who washes just as far back as his ears leaving the dirty tide mark beyond. I cannot think of a better simile—better by far would it have been had we devoted the fifteen or twenty minutes to the care of those neglected parts in the mouth and let the self-cleansing surfaces take care of themselves.

How long do we take to give a prophylaxis treatment? Usually two hours, if we cover the whole mouth at the first operation. I find it is better policy, however, not to go over the whole mouth at the first sitting; take a half only, and if there is tenderness as a result of the first treatment, it is better to have the half mouth tender rather than the whole. Not that alone, but you will get a better fee for your work—a patient is much more willing to pay you an adequate sum for your labor when it consists of *two* operations, even if it takes up the same amount of time than for one long operation. This brings me to the fee question; this work must be charged for by the hour at the same rate as your other services; you must not charge less, you will belittle it for prophylaxis is infinitely more valuable to him than restoration. You will please not speak of it as cleaning teeth, for "cleaning teeth" conveys something so radically different to the patient's idea, from what a prophylaxis treatment really is, you will explain the meaning of the word prophylaxis to your patient at the first operation that it is a compound word made up of two Greek words—phylasso, to guard, prophylasso to keep guard before used by the ancient Greeks relative to the soldiers keeping guard over the entrances to the city—the outposts.

You will make that first operation such an interesting one that your patient is lost in wonder, at each turn he learns something new. You use no engine work in any part of the process, for by so doing you defeat the object, for it is by a cultivated sense of touch that we discern what we are doing, an engine reveals nothing to the tactile sense; it is a case of feeling and not seeing; you will give your patien such splendid instructions in the care of his mouth that he will place unlimited confidence in you and will feel that he has in you a true friend, inasmuch as you are taking the greatest pains in order to show him how to *prevent* decay; you will have every mother as she leaves your office arranging for an appointmen for Mary and Tommy, and at her next visit her husband, she says, would like to have you go over his teeth in just the same manner. Aunt Mary, yes, and grandma, if she has one tooth left, looks upon you as a godsend, and all, the whole family, want your services and want them quickly.

You will have your patient at the second visit look at you wistfully to see if you approve of what he has been doing for his mouth in the interval, and you praise, where you can; but the delinquent spots (which your eagle eye catches immediately on noting the morbid color of the gum), are condemned, and the patient feels he is at fault. At the third visit you have him bring his tooth brush and go through a sort of washbowl clinic at the office; he is amazed at how much you still have to show him and correct him in. It is not a question of brushing *teeth*, not at all, it is brushing *gums*. I tell my patients that they need not fear, their teeth will not be neglected as they get taken care of in the tail end of the stroke, the idea is to have the free margins of the gums cared for where so much infection lies. The manipulation of the brush is known as the rolling movement; the *correct* way to use floss ribbon is shown, wrongly used it does more harm than good, rightly used it plays a much greater part in the prevention of decay than does the brush relative to the approximal surfaces. In giving instructions to patients and intimating what you require of them, one has to be very autocratic in one's demands—the patient's co-operation in prevention is absolutely essential. You don't disguise the fact from him, either; he learns that it is possible only to have immunity by conscientious work on *his* part, as well as that of yours. Both night and morning care must be given in a scientific manner. Almost anyone can care for his mouth twice a day; where recession of gums is in progress this must be stopped at once and the tissues brought back to place by correct massage movements—it gives one the very greatest pleasure to watch the restoration of the gum tissues—to recreate those beautiful festoons. The patient was responsible for driving them away in the first place. Cross brushing cannot be condemned too emphatically; it is disastrous to gums and teeth both; the error of it is written so plainly across the teeth of almost every patient who presents himself for treatment; those deep grooves across the labial and buccal surfaces of the teeth—do you sometime question what brought them? Nothing but cross brushing. The brush started the groove, the lactic acid finding the groove lodges there, decalcifying the structure, continually making it deeper until the part has to be filled. The instruction the patient receives is quite an educative process for him, and please remember that none of this good advice, instruction and admonition is gratis; it is charged for by time, and it's wonderful how intelligent and apt he becomes in a very short while in the care of his mouth. I wish it were possible to give each one of you present a prophylaxis treatment in order that you might realize, personally, how delightful the mouth feels after such an operation.

A few words on technique, perhaps, is fitting, though practical presentation of the operation is infinitely more satisfactory. I use principally Dr. D. D. Smith's set of files for the instrumentation which is the first stage of the work; next, wooden points are carried in portepolishers for the polishing process; don't use ordinary pumice, it's too coarse; use instead a flour of pumice put up by the Buffalo Dental Mfg. Co. This produces a verty brilliant lustre; the polishing must be done *with* the axis of the tooth, next dental ribbon (not ordinary dental floss) is drawn through interproximal spaces, polishing two walls thoroughly in every space, not by any means an aimless pulling through, for these spaces are the vulnerable parts upon which we must dwell longer than any other surface of the tooth; following all of this must come the atomizers using anywhere from 40 to 60 pounds of pressure compressed air. Compressed air can be installed at very moderate cost. A water pump or an electric pump may be used, and a tank, a container which will hold 12 gallons may be had for $12 or $14. The spraying is a very essential part of the process, washing the debris from pockets and around margins in the most effectual way; it is that stage of the operation which the patient hails with delight. One last operative movement and the operation is done. This is the massage. I apply this as an object lesson only at the first treatment for after that the patient soon becomes expert at it himself. Massage along corrcet lines accomplishes so much. The utmost thoroughness is absolutely essential in this work. Without thoroughness we cannot accomplish prophylaxis prevention, we shall instead bring ignominy on ourselves, on our promises, and on the fair name of prophylaxis. It is not easy work, it is arduous, painstaking and exacting. The strain upon one's hand is very great, i. e., on a certain set of fingermuscles, but the glorious results bring their own reward. The usual expression of a patient as he leaves the chair is to the effect that his mouth feels perfectly delightful. Just one word on pyorrhea and then I must draw to a close. Sometimes one finds it hard to decide whether or not it is justifiable to extract, where the teeth seem to be pretty loose. A good rule to go by is, should you get a rotary movement of tooth, condemn it to the forceps without hesitation; if only a lateral movement, proceed to give treatment, with the object of restoring. The rotary movement denotes loss of bone structure and terminal bone structure cannot be reconstructed, while it is a comparatively easy matter to reproduce gum tissue.

We all know, of course, who is the father and author of our present systematized prophylaxis,—Dr. D. D. Smith of Philadelphia. I was honored by an invitation from Dr. Smith five years ago to come and see what he had to show us. I went from Boston along with five other practitioners, and during those two days' sitting at the master's feet, I saw upwards of forty of the most beautiful mouths I had ever looked upon, dreams of loveliness. Dr. Smith's patients come when he bids them, so grateful are they to him. I tell you, one cannot look in upon a prophylaxis practice and see such a glorious sight without coming away with the desire, no, the determination to give prophylaxis the first rank in one's practice. I confess to you that six years ago at the beginning of my interest in the work, previous to my going to Dr. Smith's office, I looked upon prophylaxis (prevention) as a sort of visionary, impractical dream, and thought that where theory ended and the practical began it could not hold its own. It was, in other words, too impracticable, it was altogether to much of idealism. But a visit to Dr. Smith's office forever silenced such thoughts and now after upwards of six years of persistent following of prophylaxis with a practice composed almost entirely of this work and pyorrhea, I have abundant proof in the many mouths to show, should you care to come, that would convince the most skeptical that we *can* and do *prevent* decay and at the same time bring about such a change in the appearance of the mouth it is beautiful to behold; a prophylaxis mouth is an everlasting joy. Compared with prophylaxis, the other kind of dentistry, *restoration*, seems to be so inadequate, so inefficient, in a way it seems as if we had missed our goal.

Prophylaxis is coming into its own. Several colleges have devoted chairs to the subject; there is scarce a large city but has either special practices devoted to this work, or else it is a department of a large practice. Prophylaxis has taken up its permanent abiding place with us. Won't you identify yourself with it?—*Dental Summary.*

